# Outcomes in Symptomatic Patients With Vertebrobasilar Dolichoectasia Following Endovascular Treatment

**DOI:** 10.3389/fneur.2019.00610

**Published:** 2019-06-13

**Authors:** Jiejun Wang, Luqiong Jia, Xinjian Yang, Xuecang Jia, Jian Liu, Peng Liu, Zefeng Miao, Ying Zhang, Zhongbin Tian, Kun Wang, Zhongxiao Wang, Yisen Zhang, Ming Lv

**Affiliations:** ^1^Beijing Neurosurgical Institute, Beijing Tiantan Hospital, Capital Medical University, Beijing, China; ^2^Taian Hospital of Traditional Chinese, Taian, China; ^3^Heping Hospital Affiliated to Changzhi Medical College, Changzhi, China

**Keywords:** vertebrobasilar dolichoectasia (VBD), symptomatic patients, endovascular treatment, vertebral artery, basilar artery

## Abstract

**Objective:** To evaluate whether the presenting symptoms of VBD predict outcomes following endovascular treatment.

**Methods:** We retrospectively reviewed our institutional clinical database and identified 22 patients (all men; mean age: 52.6 years, range: 11–73 years) with a diagnosis of VBD, who underwent endovascular treatment from January 2010 to December 2017.

**Results:** After analyzing the clinical and imaging data, we evaluated data for 22 symptomatic patients with VBD. At the time of VBD diagnosis, 13 patients (59%) had compressive symptoms, four (18%) had hemorrhagic symptoms, and five (23%) had ischemic symptoms. Nine of the 22 patients (41%), who presented with hemorrhagic and ischemic symptoms, achieved a satisfactory clinical and/or digital subtraction angiography imaging outcome after endovascular treatment. However, of the 13 patients who presented with compressive symptoms, seven (54%, 7/13) died from severe brainstem compression during follow-up; furthermore, magnetic resonance imaging showed worsening of the mass effect in eight patients with compressive symptoms (62%, 8/13).

**Conclusions:** VBD is considered a challenging lesion without an ideal treatment modality. Endovascular treatment of VBD in patients presenting with compressive symptoms at diagnosis may not be beneficial. However, long-term outcomes following endovascular treatment may be acceptable in patients with non-compressive symptoms at diagnosis compared with those with compressive symptoms.

## Introduction

Vertebrobasilar dolichoectasia (VBD) is characterized by dilatation, elongation, and tortuosity of the vertebrobasilar arteries, with the presenting symptoms resulting from ischemia or compression of the brainstem. Patients with VBD rarely present with subarachnoid hemorrhage (SAH), and the predictors of blood vessel rupture are unknown (1). Dolichoectasia is associated with hypertension, older age, and male sex; it is also reportedly associated with heritable connective tissue disorders such as Marfan syndrome and Ehlers–Danlos syndrome (2). The diagnostic criteria for VBD on computed tomography (CT) were developed in 1986, and those on magnetic resonance imaging (MRI) were developed in 1988 (3–5).

VBD is a challenging lesion without an ideal treatment modality, and it usually carries a poor prognosis. The estimated 5-year mortality of patients with VBD is 36.2%, and the 5-year prognosis is more favorable in patients who are asymptomatic at the time of diagnosis (6). For some asymptomatic patients, the attending neurologists and radiologists may choose conservative therapy after performing a risk-benefit evaluation. However, there are currently no effective treatments for symptomatic patients with ischemia, hemorrhage, and/or compression (2). The treatment of VBD via endovascular reconstruction using stent-assisted coiling (SAC) or sole stents in the vascular lumen is technically feasible, and is relatively safe compared with the poor natural disease course of VBD (7). The present study aimed to evaluate the role of the initial symptoms in symptomatic patients with VBD in predicting outcomes after endovascular treatment.

## Methods

### Study Design

This was a retrospective review of our experience treating VBD using endovascular treatment. The study was approved by the ethics committee of Beijing Tiantan hospital and met the guidelines of the Declaration of Helsinki. Written informed consent for study inclusion and publication was obtained from the patients themselves or from their relatives.

### Patients

We retrospectively reviewed our institutional clinical database (Beijing Tiantan Hospital, Beijing, China) and identified 4,720 patients diagnosed with intracranial aneurysms from January 2010 to December 2017, including 488 patients with intracranial aneurysms located in the vertebrobasilar system. From these patients, we selected 22 patients who were diagnosed with VBD in accordance with the diagnostic criteria for VBD on CT or MRI (3–5), and these patients underwent endovascular treatment. We excluded patients diagnosed with VBD incidentally and those who were symptomatic but did not undergo endovascular treatment. The study population comprised 22 men and no women (male: female ratio, 22:0), with a median age of 52.6 years (range, 11–73 years; standard deviation, 12.6 years). All 22 patients (100%) were symptomatic, including 13 patients (59%) who presented with compressive symptoms, four (18%) who presented with hemorrhagic symptoms, and five (23%) who presented with ischemic symptoms; these symptoms were validated on preoperative MRI or CT. In detail, patients presenting with non-specific symptoms or specific symptoms associated with a mass effect, such as intracranial nerve palsy and hydrocephalus resulting from obstruction of the fourth ventricle, and with preoperative CT/MRI implying a mass effect, were enrolled in the compressive group. Ischemic patients presenting with transient ischemic attack (TIA) or non-specific symptoms and with preoperative CT/MRI implying intracranial infarction associated with lesions were enrolled in the ischemic group. Hemorrhagic patients usually presented with sudden severe headache, and with preoperative CT implying SAH, these patients were enrolled in the hemorrhagic group. We excluded patients with traumatic aneurysms and collagen vascular disorders. No patients had a known family history of VBD. Patients' demographics and clinical characteristics were collected from patients' medical records and are shown in Table 1.

**Table 1:** Characteristics, treatment, and outcomes of patients with vertebrobasilar dolichoectasia (VBD).

								**Long-term angiographic follow up**		**Clinical outcome**		
**Case**	**Age**	**Hypertension**	**Diabetes**	**Presenting symptoms**	**VBD location**	**Treatment mode**	**Immediate angiography**	**DSA**	**MRI**	**Pre-mRS**	**F/U-mRS**	^*^F/U period/m
1	44–46	Yes	No	Sudden severe headache (H)	LVA+BA	4×Solitaire+Coils	GRC+ACC	GRC+ACC	S	1	0	21
2	52–54	Yes	No	Sudden severe headache accompanying with nausea and vomit (H)	DVA+BA	2×LVIS+2×Solitaire+Coils	GRC+ACC	GRC+ACC	S	1	0	6
3	58–60	Yes	No	Chronic headache (C)	LVA+BA	3×Enterprise+Coils	GRC+AIC	GRC+ACC	E^2^	1	6	12
4	44–46	Yes	No	diminished visual acuity accompanying with dizziness (C)	DVA+BA	4×Solitaire	GRC	GRC	E^2^	2	6	29
5	48–50	Yes	No	Sudden dizziness and left limbs weakness (C)	DVA+BA	4×Solitaire	GRC	E^1^	E^2^	2	6	48
6	50–52	Yes	No	Sudden faint and consciousness loss (I)	BA	2×Lvis	GRC	GRC	S	2	0	34
7	54–56	Yes	No	diminished visual acuity and diplopia (I)	BA	2×Lvis+1×Solitaire	GRC	GRC	S	2	1	96
8	38–40	No	No	Sudden dizziness and consciousness loss (I)	BA	2×Enterprise+1×Neuroform	GRC	GRC	NA	5	5	78
9	58–60	Yes	No	Chronic dizziness (I)	LVA+BA	2×Pipeline+Coils	GRC+AIC	GRC+ACC	S	1	0	6
10	40–42	Yes	No	Sudden faint accompanying with severe headache (H)	RVA+BA	2×Enterprise+Coils	GRC+ACC	GRC+ACC	NA	1	1	83
11	64–66	Yes	No	Chronic headache (C)	RVA+BA	2×Enterprise+Coils	GRC+AIC	GRC+ACC	NA	1	6	42
12	72–74	Yes	No	Trigeminal neuralgia (C)	RVA+BA	2×Enterprise+Coils	GRC+AIC	GRC+ACC	E^2^	2	0	58
13	52–54	Yes	Yes	Chronic dizziness (C)	LVA+BA	3×Enterprise+Coils	GRC+AIC	E^1^	E^2^	1	6	42
14	62–64	Yes	No	Chronic headache and hearing loss (C)	LVA+BA	1×Enterprise+Coils	GRC+AIC	GRC+ACC	NA	1	1	45
15	56–58	Yes	No	Right limbs hemiparesis (I)	LVA+BA	1×Enterprise+Coils	GRC+AIC	GRC+ACC	S	1	1	44
16	60–62	Yes	Yes	Headache and diminished visual acuity (C)	LVA+BA	2×Enterprise	GRC	GRC	NA	2	6	27
17	44–46	Yes	No	Quadriplegia and consciousness loss (C)	RVA+BA	4×Solitaire	GRC	GRC	E^2^	5	6	72
18	10–12	No	No	Chronic headache (C)	BA	PAO	PAO	PAO	NA	2	1	30
19	60–62	Yes	No	Headache and lower limbs weakness (H)	LVA+BA	2×Enterprise+Coils	GRC+AIC	GRC+ACC	S	2	1	78
20	54–56	Yes	No	Chronic headache (C)	DVA+BA	3×Enterprise	GRC	GRC	NA	1	0	78
21	54–56	Yes	No	Right facial palsy (C)	LVA+BA	1×LEO	GRC	GRC	E^2^	1	2	85
22	64–66	Yes	Yes	Right limb paresis and dysphagia (C)	DVA+BA	3×Solitaire	GRC	GRC	E^2^	2	3	78

H, hemorrhagic symptoms; C, compressive symptoms; I, ischemic symptoms; LVA, left vertebral artery; BA, basilar artery; DVA, dual vertebral artery; RVA, right vertebral artery; WRC, good reconstruction of the diseased artery; ACC, complete occlusion of the aneurysm; AIC, incomplete occlusion of the aneurysm; PAO, parent artery occlusion; DSA, digital subtraction angiography; E^1^, enlargement of the diameter of the diseased artery; E^2^, enlargement of the mass effect; S, follow-up imaging unchanged compared with preoperatively; NA, not available;

* *F/U period, time after endovascular treatment (in months)*.

### Treatment Selection

We selected patients with ischemic and compressive symptoms who did not obtain satisfactory improvement of their clinical symptoms after undergoing conservative treatment, namely, antiplatelet therapy, oral anticoagulants, and symptomatic treatment. In patients presenting with a mass effect, we chose either multiple stenting or flow diversion, and we placed flow-diverting stents in an attempt to divert flow away from the enlarged lumen and reduce the size of the vessel over time. Our goal was to slow aneurysmal growth or diminish the mass effect secondary to reduced pulsatile blood flow to alleviate the symptoms arising secondary to the mass effect, such as headache, cranial nerve palsy, and hydrocephalus. In patients presenting with ischemic symptoms, given the complexity of the lesions, antiplatelet therapy or oral anticoagulants were considered the first choice to alleviate related symptoms such as dizziness, hemiparalysis, and TIA. If patients showed no clinical improvement, endovascular treatment was a therapeutic choice to tack down the torn endothelial tissue, remodel the vessel, and reduce the chance of microthrombus formation to prevent further progression of the intracranial infarction area. In patients with hemorrhagic symptoms, medical treatment was not our first choice to control intracranial hemorrhage by excluding lesions; furthermore, neurosurgery was considered unlikely to achieve a satisfactory outcome, and to carry a high degree of risk because of the special anatomical structures around the brainstem. Therefore, these patients underwent endovascular treatment, including sole stents (45%, 10/22), SAC (50%, 11/22), and parent artery occlusion (PAO; 5%, 1/22).

### Endovascular Procedures

All patients underwent endovascular treatment under general anesthesia, and received systemic heparinization after sheath placement. A 6F Envoy® guiding catheter (Cordis, Miami Lakes, FL, USA) was navigated into the C1–C2 level vertebral artery. According to the measured length and diameter of the diseased target artery, we inserted multiple Enterprise® stents (Codman, Raynham, MA, USA), Solitaire® stents (ev3, Irvine, CA, USA), low-profile visible intraluminal stents (Microvention, Tustin, CA, USA), Neuroform® stents (Stryker, Kalamazoo, MI, USA), LEO® stents (Balt Extrusion, Montmorency, France), or pipeline embolization devices (PED) (Medtronic, Dublin, Ireland). We initially used conventional stents to treat patients with VBD, until the development of PEDs with a greater metal coverage rate (30%−35%) in blood vessels. It was crucial that the distal portion of the stent was placed in the normal portion of the distal target vessel. We generally chose the overlapping stent technique to reconstruct the lumen of the diseased arteries, and ensured that the proximal portion of the stent covered the proximal normal portion of the target artery. Stents were placed telescopically in long arterial segments that required bridging. If aneurysms or large eccentric lumens originated from the diseased arteries, we used the jailing technique to coil the aneurysms and large eccentric lumens with the assistance of stents. PAO was performed in patients with particularly severe VBD in whom the balloon occlusion test indicated adequate collateral circulation from the anterior circulation.

### Antiplatelet and Anticoagulation Treatments

Patients without hemorrhage were premedicated with a dual antiplatelet regimen (75 mg of clopidogrel and 100 mg of aspirin daily) for 5 days before treatment. Patients with hemorrhage received a loading dose of 300 mg aspirin and 300 mg clopidogrel 2 h preoperatively. Intraoperatively, we administered an intravenous bolus dose of heparin (100 IU/kg), and continued heparinization to maintain an activated clotting time throughout the procedure of two to three times greater than the baseline value. Dual antiplatelet therapy was continued for 6 months post-operatively, and aspirin was continued for life to prevent thrombi forming in the stents.

## Results

Of the 22 patients, 13 patients (59%) presented with compressive symptoms, four (18%) presented with hemorrhagic symptoms, and five (23%) presented with ischemic symptoms. The definitive initial symptoms and the modified Rankin score (mRS) of each patient are summarized in Table 1. SAC was used in 11 patients (50%, 11/22), sole stents were used in 10 (45%, 10/22), and PAO was used in one (5%, 1/22). All patients tolerated the procedure well except one patient (case 2, 5%, 1/22) with intraoperative complications. Digital subtraction angiography (DSA) performed immediately after the procedure showed good reconstruction of the diseased arteries in all patients.

### Follow-Up of the Patients With SAH

Clinical and/or imaging follow-up was available for a mean period of 49.6 ± 27.7 months. Four patients (18%) with SAH achieved satisfactory clinical and angiographic outcomes; MRI follow-up was available for three patients (75%, 3/4), which demonstrated no change compared with preoperative MRI.

### Follow-Up of the Patients With Compression

The 13 patients (59%) with compressive symptoms had worse outcomes than the patients with hemorrhagic and ischemic symptoms. Seven patients (54%, 7/13) died from severe brainstem compression during follow-up (mean, 38.9 months; range, 12–72 months), and follow-up MRI performed in five patients (71%, 5/7) demonstrated worsening of the mass effect. Clinical symptoms deteriorated in two patients (15%, 2/13), were unchanged in one patient (8%, 1/13), and showed mild improvement in three patients (23%, 3/13). Follow-up DSA showed that the diameter of the diseased artery was unchanged in 11 patients (85%, 11/13), and enlarged in two patients (15%, 2/13). MRI follow-up was available for eight patients (62%, 8/13), and confirmed worsening of the mass effect to varying degrees.

### Follow-Up of the Patients With Ischemia

Of the five patients with ischemic symptoms, the clinical symptoms improved in three patients (60%, 3/5), while two patients (40%, 2/5) had no change in their clinical symptoms during follow-up. Specifically, during the follow-up of ischemic patients presenting with TIA, if the occurrence rate of TIA was lower than the preoperative rate, or no recurrent strokes occurred by the final follow-up, patients' clinical outcomes were considered improved if they had a low mRS score. For ischemic patients presenting with chronic symptoms secondary to intracranial infarction, if patients' symptoms improved and they had a low mRS score, their clinical outcomes were considered improved. DSA follow-up data were available for all five patients with ischemic symptoms (100%, 5/5) and showed no changes (including reduction or enlargement) in the diameters of the diseased arteries. MRI follow-up data were available in four patients with ischemic symptoms (80%, 4/5) and demonstrated no deterioration of the infarction area compared with preoperative MRI.

Patients' clinical and imaging follow-up results are summarized in Table 1.

## Case Examples

### Case 2

**Figure 1:**
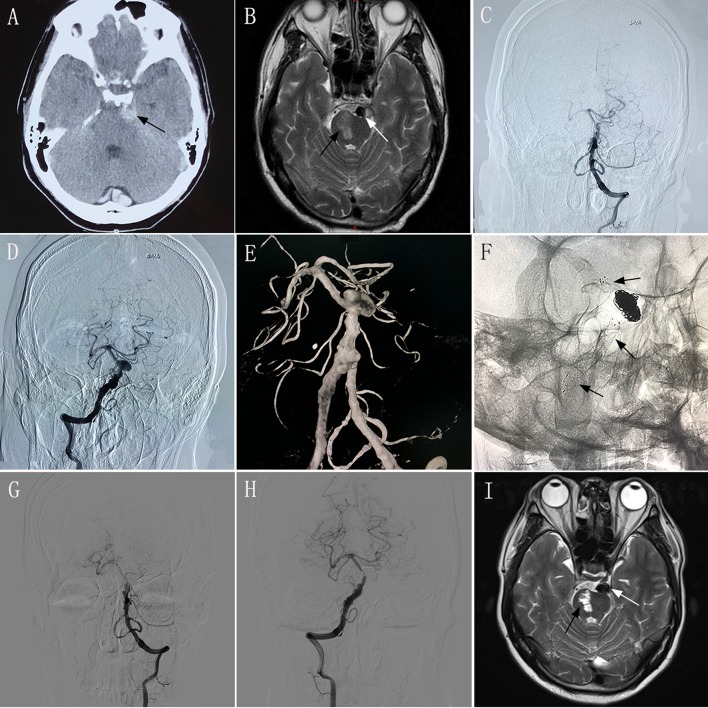
Images from a 52–54-year-old man with vertebrobasilar dolichoectasia (VBD) involving the dual vertebral artery (DVA) and basilar artery (BA) (case 2). **(A)** Computed tomography performed at another hospital showing subarachnoid hemorrhage around the brainstem (black arrow). **(B)** Preoperative magnetic resonance imaging (MRI) showing the diseased artery (white arrow) and left brainstem infarction (black arrow). **(C,D)**: Preoperative anteroposterior angiogram of the left vertebral artery **(C)** and the right vertebral artery **(D)** showing that the VBD involved the DVA and basilar artery. **(E)** Preoperative three-dimensional reconstruction confirming the involvement of the DVA and basilar artery. **(F)** Intraprocedural unsubtracted view showing the location of the two low-profile visible intraluminal stents (4.5 × 30 mm and 5.5 × 25 mm), two Solitaire® stents (both 6 × 30 mm), and coils. **(G,H)** 6 month post-treatment anteroposterior angiograms of the left VA **(G)** and right VA **(H)** showing patency of the diseased artery and complete occlusion of the aneurysm. **(I)** 6 month post-treatment MRI showing good reconstruction of the lumen (white arrow) and stability of the left brainstem infarction (black arrow) compared with preoperative MRI.

A 52–54-year-old man experienced sudden severe headache accompanied by nausea and vomiting for 2 days, and had an mRS score of 1. CT performed at another hospital showed SAH (Figure 1A), and the patient was transferred to our hospital. MRI performed in our hospital showed a left brainstem infarction (Figure 1B). Based on the diagnostic criteria for VBD on CT and MRI (3–5), the neurologist and radiologist diagnosed this patient has having VBD. Given the complexity of the lesion, endovascular treatment using the overlapping stent technique was considered a feasible treatment modality. The intraprocedural DSA images confirmed the diagnosis of VBD (Figures 1C–E). Two low-profile visible intraluminal stents (4.5 × 30 mm and 5.5 × 25 mm) and two Solitaire® stents (both 6 × 30 mm) were inserted into the diseased artery (Figure 1F). DSA performed immediately post-operatively revealed good reconstruction of the basilar artery, and dense embolization with coils in the aneurysm. However, after inserting the stents, slow blood flow was observed in the basilar artery according to immediate post-procedural angiography. We immediately administered tirofiban, including a loading dose of 0.6 mg by intravenous injection and 0.25 mg/h by constant-rate intravenous infusion until noon of the second post-operative day. After undergoing treatment with tirofiban, blood flow velocity in the patient's basilar artery normalized. Following the endovascular procedure, this patient developed no new symptoms compared with his preoperative condition. During 6 months of post-treatment follow-up, his clinical symptoms completely resolved, providing an mRS score of 0. Follow-up imaging showed good reconstruction of the basilar artery and complete occlusion of the aneurysm on DSA (Figures 1G,H), and no change in the area of brainstem infarction on MRI (Figure 1I) compared with preoperative MRI.

### Case 5

**Figure 2:**
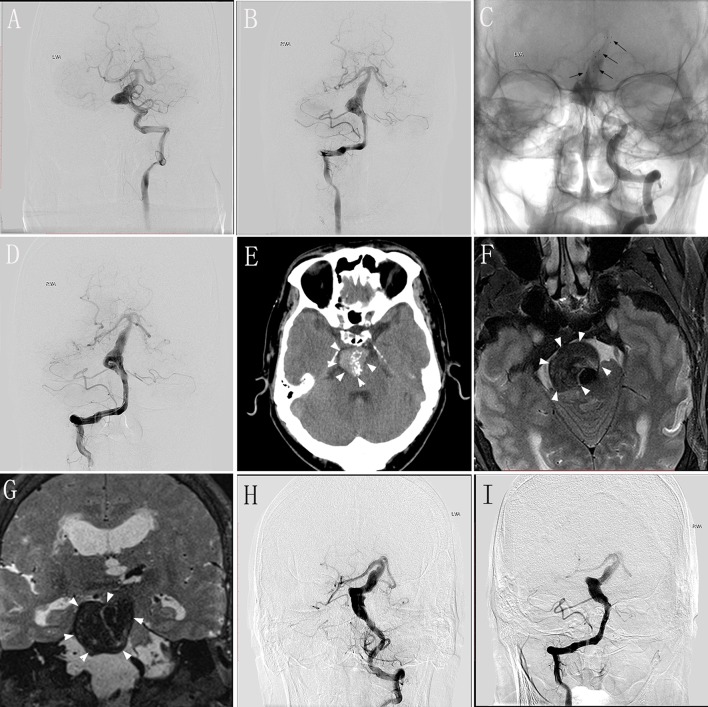
Images from a 48–50-year-old man with vertebrobasilar dolichoectasia (VBD) involving the basilar artery (BA) (case 5). **(A,B)** Preoperative digital subtraction angiography (DSA) of the left vertebral artery (VA) **(A)** and right VA **(B)** showing the involvement of the BA. **(C)** Unsubtracted view of the left VA immediately post-treatment showing the location of the stents (black arrows). **(D)** Immediately-post-treatment angiography of the right VA showing no change in the diameter of the diseased artery and obvious stasis of the contrast agent. **(E)** Follow-up computed tomography 5 months post-treatment showing a mass effect with compression of the brainstem (white arrowheads). **(F,G)** Transverse **(F)** and coronal **(G)** magnetic resonance images 24 months post-treatment showing severe brainstem compression. **(H,I)** Follow-up DSA of the left VA **(H)** and right VA **(I)** at 24 months showing the deterioration in the length and diameter of the diseased artery.

A 48–50-year-old man experienced dizziness and weakness in his limbs for 1 month, and had an mRS score of 2. MRI and DSA performed in our hospital validated the diagnosis of VBD involving the basilar artery (Figures 2A,B). We performed endovascular treatment with the overlapping stent technique to reconstruct the lumen of the basilar artery. Four Solitaire® stents (three 6 × 30 mm and one 6 × 20 mm) were inserted into the basilar artery. DSA performed immediately post-operatively revealed no change in the diameter of the basilar artery with obvious stasis of the contrast agent (Figures 2C,D). The patient underwent clinical and/or imaging follow-up examinations at 5, 17, 24, and 48 months after the procedure. At 5 months during follow-up, the patient underwent repeat DSA, which showed no change compared with immediately-post-operative angiography, and he had no obvious symptoms. At 17 months during follow-up, he underwent DSA again, which showed deterioration compared with the DSA performed 5 months after the procedure, although he continued to have no obvious symptoms. At 24 months during the follow-up, the patient presented with headache and diminished visual acuity. Follow-up CT at 5 months (Figure 2E) and MRI at 24 months (Figures 2F,G) demonstrated severe compression of the brainstem with dramatic progression. DSA follow-up at 24 months showed that the length and diameter of the diseased artery had deteriorated (Figures 2H,I). Relatives confirmed that this patient died from pneumonia secondary to severe brainstem compression 48 months post-operatively.

### Case 9

**Figure 3:**
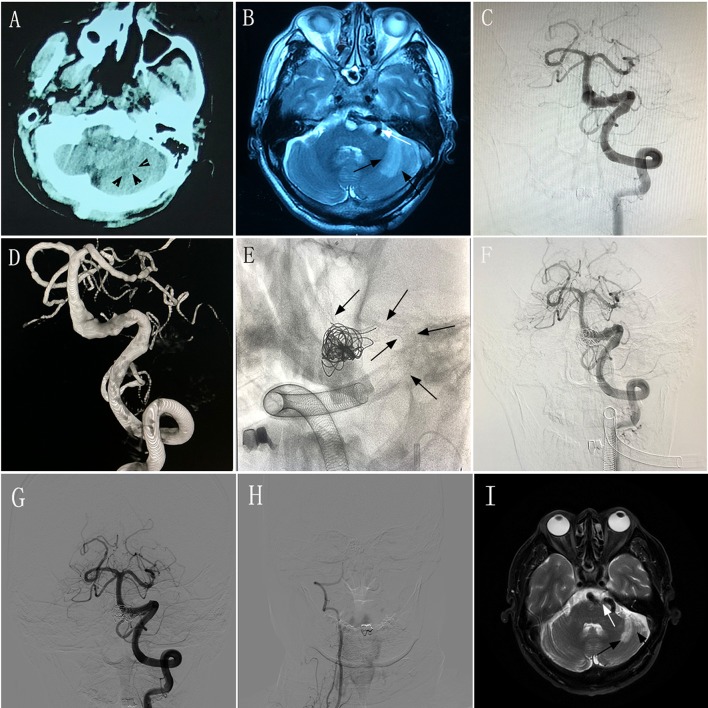
Images from a 58–60-year-old man with vertebrobasilar dolichoectasia (VBD) involving the left vertebral artery (VA) and basilar artery (BA) (case 9). **(A)** Computed tomography performed at a local hospital showing a hypodense area in the left cerebellum (black arrowheads). **(B)** Magnetic resonance imaging (MRI) performed at a local hospital confirming left cerebellar infarction (black arrows) and a tortuous diseased artery (white arrow). **(C,D)** Preoperative anteroposterior angiogram **(C)** and three-dimensional reconstruction **(D)** of the left VA demonstrating that the VBD involved the left VA and BA. **(E)** Intraprocedural unsubtracted view showing the location of two pipeline embolization devices (4.75 × 35 mm and 5.0 × 35 mm) and coils (black arrows). **(F)** Immediately-post-treatment angiogram showing the patency of the diseased artery. **(G)** Anteroposterior angiogram of the left VA 6 months post-treatment showing good construction of the lumen of the diseased arteries. **(H)** Anteroposterior angiogram of the right VA 6 months post-treatment showing complete occlusion. **(I)** MRI at 6 months post-treatment showing the tortuous diseased artery (white arrow) and stable infarction area in the left cerebellum (black arrows) compared with preoperative MRI.

A 58–60-year-old man experienced intermittent dizziness for 1 month, accompanied by occasional nausea and vomiting. CT, MRI, and magnetic resonance angiography performed at a local hospital showed left cerebellar infarction, and VBD involving the left vertebral artery and basilar artery (Figures 3A,B). The patient had an mRS score of 1. After transferring to our hospital, the patient underwent DSA, which confirmed the diagnosis of VBD (Figures 3C,D) and occlusion of the right vertebral artery. The neurologists and radiologists opted to perform endovascular treatment with the overlapping stent technique. Two PEDs (4.75 × 35 mm and 5.0 × 35 mm) were inserted into the basilar artery and left vertebral artery (Figure 3E) and we used adjunctive coils to occlude the distal portion of the right vertebral artery. DSA performed immediately post-operatively showed good reconstruction of the diseased artery (Figure 3F). During 6 months of post-treatment follow-up, the patient had a good clinical outcome with an mRS score of 0. Imaging showed that the diseased arteries were well-reconstructed, with complete occlusion of the right vertebral artery (Figures 3G,H), and there was no change in the infarction area of the left cerebellum (Figure 3I) compared with preoperative MRI and DSA.

## Discussion

Patients with VBD have a high risk of ischemic stroke, brainstem compression, and death, while hemorrhagic complications are less common (6). A previous systematic review of VBD concluded that the risk of bias in the included studies was too high to make any recommendations regarding treatment. However, asymptomatic patients have a more favorable course than patients who are already symptomatic at the time of diagnosis (6). Thus, conservative management may be reasonable in asymptomatic older adult patients. However, patients with VBD had a high risk of neurological deterioration during 5 years of follow-up, in one study (6). Considering this poor natural history, intervention should be considered in symptomatic patients. With modern endovascular techniques and devices, reconstruction of large and giant dolichoectatic aneurysms is feasible (8). Some studies have reported using endovascular treatment for VBD (7–12); however, outcomes varied, and although some patients reportedly achieved good outcomes after endovascular treatment, the majority of studies were case reports with bias regarding patient selection. Patients' clinical outcomes depend on the characteristics and locations of the aneurysms, as well as the presenting symptoms (10). Our study revealed the important role of the initial symptoms in predicting the prognosis of symptomatic patients with VBD after endovascular treatment.

### Patients With Compressive Symptoms

Patients with VBD may present with subacute to chronic symptoms related to the compression of adjacent structures. Compression of surrounding structures in the vertebrobasilar system may result in brainstem compressive syndromes, such as single and multiple cranial neuropathies (typically involving cranial nerves 5, 6, 7, and 8) and hydrocephalus (13). Compression may be gradual, allowing for adaptation, which can explain the poor correlation between clinical symptoms and the extent and severity of compression (14), or VBD may be progressive (15, 16). A recent study evaluating the natural disease course of VBD reported progression in nearly half (16/35) of the patients who presented with compression; additionally, 12 patients (7.5%) who did not have compressive symptoms at the initial diagnosis developed new compressive symptoms (13). Therefore, the frequent presence of a mass effect on the brainstem often necessitates treatment rather than observation (11). Little is known about the long-term treatment outcomes in patients with a mass effect on the brainstem (8). VBD may continue to grow even after occlusion of the proximal portions of the bilateral vertebral arteries (17). In our study, worsening of the mass effect was observed in eight patients with compressive symptoms (62%, 8/13), and seven patients (54%, 7/13) died from severe brainstem compression during follow-up.

The following factors may contribute to the progression of the mass effect: (1) acute intramural hemorrhage may promote VBD development and growth (18); (2) multilayer acute-on-chronic thrombi indicates progressive thrombosis secondary to disturbed blood flow (19); (3) neovascularization of the thrombus in giant aneurysms contributes to recanalization (20); and (4) embolic materials take up space within an aneurysmal sac, which may limit shrinkage (12). Additionally, basilar aneurysms that present with symptoms of a mass effect on the brainstem and cranial nerves have a tendency to progressively increase to an ultimately fatal size (8), as seen in our study.

### Patients With Ischemic Symptoms

The most common presentation in symptomatic VBD is ischemic symptoms, usually affecting the brainstem, and most often located in the pons (21, 22). Patients with VBD are also prone to ischemic stroke and coronary artery disease (21, 23). Furthermore, in patients with VBD, the risk of brain ischemia is clearly greater than that of patients with intracranial hemorrhage (24). The proposed mechanisms of cerebral infarction in patients with VBD include low flow, thromboembolism, dissection, or compression of perforating vessels (14, 22, 25); another potential mechanism is that elongation and tortuosity of the vertebrobasilar arteries can stretch the perforating branches, leading to reduced flow (23, 26). Patients with VBD have a high risk of recurrent ischemic stroke (22, 27), and radiological progression of VBD contributes to the higher risk of ischemic stroke. Stroke events in patients with VBD are associated with hypertension, the severity of basilar artery elongation, and atherosclerotic changes in the posterior circulation (21, 28). In addition, dolichoectasia is an arteriopathy rather than an atherosclerosis; thus, antiplatelet or anticoagulant therapy is not as effective for VBD as for atherosclerotic or embolic infarction, and increases the potential for VBD rupture (29). There are currently no effective measures to manage patients with VBD with ischemic symptoms. However, in our study, five patients (23%, 5/22) with ischemic symptoms underwent endovascular treatment and achieved good reconstruction of the diseased arteries. Furthermore, clinical symptoms improved after treatment in three of these five patients (60%, 3/5), and did not deteriorate in the remaining two (40%, 2/5).

### Patients With SAH

In addition to compressive symptoms and ischemia, patients with VBD aneurysms may also present with SAH, which is heralded by the usual symptoms of acute headache and focal neurological symptoms. Previous studies have reported that patients with VBD experience an unexpectedly high frequency of intracranial hemorrhage; however, separate analyses for SAH were not performed because of the small numbers of patients (30, 31). Hemorrhage might result from damage to vascular beds caused by VBD coupled with hypertensive atherosclerotic degeneration, causing progressive arteriolar wall damage (24). Furthermore, pathological changes in the arterial wall, consisting primarily of defects in the internal elastic lamina with thinning of the media secondary to smooth muscle atrophy, may predispose patients to various types of intracranial hemorrhage (24). Intracranial hemorrhage has been correlated with a larger basilar artery diameter and greater basilar artery shift (24). Compared with other SAH etiologies, mortality from SAH is twice as high in patients with VBD (19). Therefore, once the hemorrhagic accident occurs, it is unclear whether the condition should be managed as an aneurysmal rupture. However, a recent study reported that none of the patients with SAH lost consciousness at onset, and the outcome was good in all but one patient who had an extensive rebleed within 24 h of onset and then died (24). In our study, all four patients with SAH had a good angiographical outcome, and three (75%, 3/4) achieved clinical improvement during follow-up.

### Therapeutic Experience in Our Center

By reviewing our experience in treating VBD, we found that patients' initial symptoms had important implications for the prognosis after endovascular treatment. Patients with compressive symptoms at diagnosis had a poorer prognosis after endovascular treatment than those with non-compressive symptoms (including hemorrhagic and ischemic symptoms). Previous studies report that the severe compression of cerebral structures (including perforating vessels) and the stretching of perforating branches secondary to elongation and tortuosity of the vertebrobasilar arteries leads to decreased blood flow supplying important cerebral tissues, and thus cerebral infarction (23, 26). In other words, patients with severe compressive symptoms may also have ischemic symptoms, which contributes to a poorer prognosis. In patients with non-compressive symptoms who undergo endovascular treatment with the overlapping stent technique to reconstruct the diseased arteries, it is possible to ameliorate the elongation and tortuosity of dolichoectatic vertebrobasilar arteries to a certain degree, and to relieve the stretching of perforating branches secondary to VBD. Furthermore, this treatment improves blood flow in the perforating branches that originate from the diseased arteries, and reduces the risk of recurrent ischemic events. In addition, the particular pathology of VBD, namely, fragmentation of the internal elastic lamina, thinning of the media, and the extent of chronic dissection, makes these vessels vulnerable to hemorrhage. Therefore, endovascular treatment with traditional stents or flow-diverting devices to reconstruct the lumen of diseased arteries may reduce the risk of death secondary to aneurysm rupture; however, the efficacy of this treatment requires further evaluation.

### Using Flow Diverters in VBD

PEDs are a type of flow diverter. The concept of flow diversion is based on a redirection of blood flow along the longitudinal vessel axis. Hemodynamically-induced thrombosis of the aneurysmal segment of the vessel and vessel remodeling are expected effects (32). Recently, flow diverters have been increasingly used to treat refractory aneurysms, including large/giant aneurysms, blister aneurysms, and dissecting aneurysms (33). Furthermore, our review of our therapeutic experience using PEDs for VBD suggests that this is a feasible alternative for treating VBD and may increase patient's survival time and improve their long-term prognosis compared with endovascular treatment with conventional stents. Several reports have evaluated flow-diverter insertion in patients with VBD (8, 34–38), and we summarized patients' details in Table 2. Patients with VBD undergoing flow-diverter treatment were primarily those with posterior circulation aneurysms; therefore, it was difficult to collect complete information on these patients, which poses a challenge when evaluating the safety and effectiveness of flow diverters to treat VBA. Based on a literature review, we concluded two key points about flow-diverter therapy: first, in treating VBD, multiple flow diverters or conventional stent insertion was popular for most neurointerventionists; second, when treating VBD with a small number of flow diverters or conventional stents, neurointerventionists occluded one side of the vertebral artery to reduce blood flow into the aneurysm. Undoubtedly, and as reported, both methods increase the chance of intracranial infarction. However, with further study, some reports discuss using flow diverters in patients with a giant mass effect located in the posterior circulation, and these patients all achieved obvious reduction of the mass effect (9, 39, 40). In summary, PEDs provide a promising treatment modality for VBD in patients presenting with either non-compressive symptoms or compressive symptoms, but the safety and effectiveness of PEDs in treating VBD must be validated in further studies with a sufficient number of patients.

**Table 2:** Literature summary of the use of the PED technique for vertebrobasilar dolichoectasia (VBD).

	**Number**	**Age (years)/sex**	**Location**	**Subgroup**	**Type of FDs**	**Number of devices used**	**Adjunctive coiling**	**Angiographic outcome at last follow up**	**MRI/CT outcome at last follow up**	**Clinical outcome at last follow up**
van Oel LI et al. (8)	1^*^	48/M	BA	C	Silk	3	Yes	N/A	Complete thrombosis of the aneurysm with unchanged aneurysm size	No improvement
	2^∧^	64/M	DVA+BA	C	Silk	3	Yes	N/A	N/A	Death
Lopes DK et al. (34)	3	73/M	LVA+BA	H	PED	6	No	N/A	No fusiform dilatation of vertebrobasilar artery	Mild gait imbalance
Bhogal et al. (35)	4	N/A	BA	C	PED	16	No	Good reconstruction of the vessel with only a small remnant aneurysm	No evidence of pontine infarction	N/A
	5	N/A	BA	N/A	PED	N/A	No	No progression of the disease	N/A	N/A
	6	N/A	BA	N/A	PED	3	No	Complete reconstruction of the vessel	N/A	N/A
	7	N/A	BA	I	PED	18	No	Patent basilar artery and good flow distally	Pontine infarction (peri-operation)	death
	8^&^	N/A	BA	C	PED	5	No	N/A	N/A	death
	9	N/A	BA	N/A	PED/9 P64	14	Yes	Good reconstruction of the vertebral and basilar artery with a decrease in the aneurysmal filling	N/A	N/A
	10	N/A	BA	C	PED	1	Yes	Good reconstruction of the basilar artery	No significant increase in the aneurysm size or mass effect	N/A
Natarajan et al. (37)	11	N/A	DVA+BA	Neck pain	PED	1	Yes	N/A	Paramedian infarction (peri-operation)	mRS score of 4
Cohen et al. (38)	12	51/M	BA	C	Silk	5	No	Significant reduction in the aneurysm lumen	Significant reduction in the aneurysm lumen	Marked improvement in cranial nerve function

*M, male; N/A, no data available; H, hemorrhagic symptoms; C, compressive symptoms; I, ischemic symptoms; LVA, left vertebral artery; BA, basilar artery; DVA, dual vertebral artery*.

* *The patient underwent endovascular treatment with one silk stent (FD) and two Leo stents*.

∧ *The patient underwent endovascular treatment with one silk stent (FD) and two Leo stents, and underwent the intraoperative complication of in-stent thrombosis, though mechanical thrombectomy was performed resulting in recanalization of the basilar artery, the patient died the same day*.

& *The patient was due to have coil occlusion of the contralateral vertebral artery but refused further treatment and later died from progressive mass effect*.

### Limitations

The present study has three limitations. First, this study involved retrospective data collection from a small sample. Second, four of the included patients with hemorrhagic symptoms were diagnosed with SAH with a low mRS score. Third, a longer follow-up is required, particularly for patients with hemorrhagic and ischemic symptoms.

### Conclusions

Symptomatic VBD has a poor natural disease history, and VBD is considered a challenging lesion without an ideal treatment modality. Endovascular treatment may not be beneficial for patients with VBD presenting with compressive symptoms at diagnosis. However, long-term outcomes following endovascular treatment may be acceptable in patients with non-compressive symptoms (including hemorrhagic and ischemic symptoms) at diagnosis compared with those with compressive symptoms. Our study's findings require confirmation in prospective studies with larger sample sizes.

## Data Availability

No datasets were generated or analyzed for this study.

## Author Contributions

JW and YisZ performed the manuscript writing. LJ, ZW, XJ, and ZT acquired the data. JL, KW, and PL analyzed and interpreted the data. ZM and YinZ checked the manuscript. ML and XY conceived and designed the research, and handled funding and supervision.

### Conflict of Interest Statement

The authors declare that the research was conducted in the absence of any commercial or financial relationships that could be construed as a potential conflict of interest.
